# Polystyrene Chain Growth Initiated from Dialkylzinc for Synthesis of Polyolefin-Polystyrene Block Copolymers

**DOI:** 10.3390/polym12030537

**Published:** 2020-03-02

**Authors:** Tae Jin Kim, Jun Won Baek, Seung Hyun Moon, Hyun Ju Lee, Kyung Lee Park, Sung Moon Bae, Jong Chul Lee, Pyung Cheon Lee, Bun Yeoul Lee

**Affiliations:** Department of Molecular Science and Technology, Ajou University, Suwon 443-749, Korea; playing3457@ajou.ac.kr (T.J.K.); btw91@ajou.ac.kr (J.W.B.); freethemoon@ajou.ac.kr (S.H.M.); hjulee4639@ajou.ac.kr (H.J.L.); rudfl93@ajou.ac.kr (K.L.P.); bsm1029@ajou.ac.kr (S.M.B.); leejc@ajou.ac.kr (J.C.L.); pclee@ajou.ac.kr (P.C.L.)

**Keywords:** polyolefin, polystyrene, block copolymer, coordinative chain transfer polymerization, initiator, organolithium, dialkylzinc

## Abstract

Polyolefins (POs) are the most abundant polymers. However, synthesis of PO-based block copolymers has only rarely been achieved. We aimed to synthesize various PO-based block copolymers by coordinative chain transfer polymerization (CCTP) followed by anionic polymerization in one-pot via conversion of the CCTP product (polyolefinyl)_2_Zn to polyolefinyl-Li. The addition of 2 equiv *t*-BuLi to (1-octyl)_2_Zn (a model compound of (polyolefinyl)_2_Zn) and selective removal or decomposition of (*t*Bu)_2_Zn by evacuation or heating at 130 °C afforded 1-octyl-Li. Attempts to convert (polyolefinyl)_2_Zn to polyolefinyl-Li were unsuccessful. However, polystyrene (PS) chains were efficiently grown from (polyolefinyl)_2_Zn; the addition of styrene monomers after treatment with *t*-BuLi and pentamethyldiethylenetriamine (PMDTA) in the presence of residual olefin monomers afforded PO-*block*-PSs. Organolithium species that might be generated in the pot of *t*-BuLi, PMDTA, and olefin monomers, i.e., [Me_2_NCH_2_CH_2_N(Me)CH_2_CH_2_N(Me)CH_2_Li, Me_2_NCH_2_CH_2_N(Me)Li·(PMDTA), pentylallyl-Li⋅(PMDTA)], as well as PhLi⋅(PMDTA), were screened as initiators to grow PS chains from (1-hexyl)_2_Zn, as well as from (polyolefinyl)_2_Zn. Pentylallyl-Li⋅(PMDTA) was the best initiator. The *M*_n_ values increased substantially after the styrene polymerization with some generation of homo-PSs (27–29%). The *M*_n_ values of the extracted homo-PS suggested that PS chains were grown mainly from polyolefinyl groups in [(polyolefinyl)_2_(pentylallyl)Zn]^−^[Li⋅(PMDTA)]^+^ formed by pentylallyl-Li⋅(PMDTA) acting onto (polyolefinyl)_2_Zn.

## 1. Introduction

The synthesis of block copolymers has been a topical issue in the field of polymer science and chemistry [1]. Conventionally, block copolymers are synthesized by controlled living anionic or radical polymerizations [2,3]. A typical example is polystyrene-*block*-polybutadiene-*block*-polystyrene (SBS), which is industrially produced by controlled living anionic polymerization at a large scale. Polyolefins (POs) are the most abundant polymers, produced with ethylene and α-olefins, at a scale of more than 120 million metric tons per year worldwide. However, PO-based block copolymers have rarely been synthesized because α-olefins cannot be polymerized by either anionic or radical initiators [4,5,6]. The lack of versatile synthetic tools has promoted the development of multistep routes for the syntheses of PO-based block copolymers [7,8,9,10,11,12,13,14,15]. For example, polystyrene-*block*-poly(ethylene-*co*-1-butene)-*block*-polystyrene is produced industrially via a two-step process: Controlled living anionic polymerization of styrene and butadiene, and subsequent hydrogenation of the resulting SBS [16]. 

**Scheme 1:**
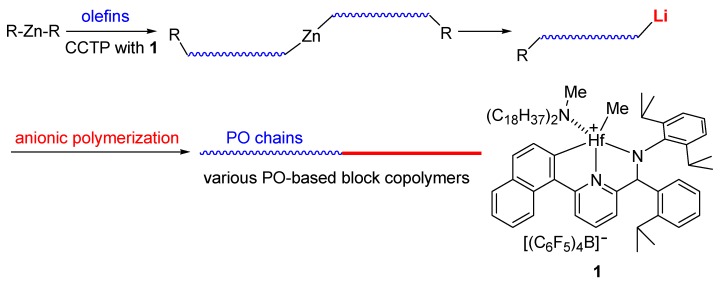
Synthetic scheme for various polyolefin-based block copolymers.

POs are mainly produced by coordination polymerization with transition-metal-based catalysts. Catalysts that can polymerize ethylene, α-olefins, or both, in a controlled living fashion, enable the synthesis of olefin block copolymers (OBCs). However, these copolymers are composed of solely PO chains with a varying ethylene/α-olefin composition [17,18,19]. An intrinsic drawback of this method is the growth of only one polymer chain per catalyst site, i.e., [PO chains]/[catalyst] = 1. A practical method for the industrial production of OBCs is based on coordinative chain transfer polymerization (CCTP). In CCTP, a transition-metal-based catalyst (e.g., 1 in Scheme 1) that can polymerize ethylene, α-olefins, or both in a controlled living fashion is used with a chain transfer agent (CTA, e.g., Et_2_Zn) in excess relative to the catalyst (e.g., [Zn]/[Hf] > 100). In CCTP, PO chains are grown uniformly and progressively from all the fed CTAs via rapid alkyl exchange between the zinc sites and the chain-growing catalyst sites [20,21]. Therefore, it is possible to grow PO chains with a varying ethylene/α-olefin composition either, by the sequential variation of ethylene/α-olefin feed ratio or by employing a dual catalytic system with distinctly different monomer reactivities. Thus, we can successfully produce diblock and multiblock copolymers composed of hard crystalline, and soft rubbery PO blocks [22,23,24,25,26]. 

(Polyolefinyl)_2_Zn results from CCTP and is usually quenched with acid to destroy the Zn-C bonds. The further growth of polymer chains initiating from (polyolefinyl)_2_Zn may be useful for the syntheses of PO-based block copolymers [27]. Syntheses of polyethylene-*block*-polyester and polyethylene-*block*-polyether have been attempted with POs functionalized with -OH end groups, which were generated by treatment of the CCTP product (polyolefinyl)_2_Zn with O_2_ [28,29,30,31]. We also discovered a method to grow polystyrene (PS) chains initiating from (polyolefinyl)_2_Zn that allows the syntheses of commercially more relevant PO-*block*-PS and PS-*block*-PO-*block*-PS in one-pot [32,33,34,35]. In those works, *n*-BuLi·(TMEDA) (TMEDA = tetramethylethylenediamine, i.e., Me_2_NCH_2_CH_2_NMe_2_) and Me_3_SiCH_2_Li·(PMDTA) (PMDTA = pentamethyldiethylenetriamine, i.e., Me_2_NCH_2_CH_2_N(Me)CH_2_CH_2_NMe_2_) were introduced to grow PS chains from (polyolefinyl)_2_Zn. In this work, we pursued a more efficient method for PS chain growth from (polyolefinyl)_2_Zn with an additional aim to expand the scope of the chains that can be grown from (polyolefinyl)_2_Zn (Scheme 1). Recently, syntheses of functionalized POs are a topical issue [36,37,38].

## 2. Materials and Methods 

All manipulations were performed under an inert atmosphere using a standard glove box and Schlenk techniques. Methylcyclohexane was purchased from Sigma-Aldrich and purified over Na/K alloy. The ethylene/propylene mixed gas was purified over trioctylaluminum (0.6 M in methylcyclohexane) in a bomb reactor (2.0 L). ^1^H NMR (600 MHz) and ^13^C NMR (150 MHz) spectra were recorded on a JEOL ECZ600 instrument. The gel permeation chromatography (GPC) data were obtained in 1,2,4-trichlorobenzene at 160 °C using a PL-GPC 220 system equipped with an RI detector and two columns [PLgel mixed-B 7.5 × 300 mm from Varian (Polymer Lab)]. (1-Octyl)_2_Zn and (1-hexyl)_2_Zn were prepared and purified as described in the literature [26]. *n*-BuLi and *sec*-BuLi were used as neat oils, while *t*-BuLi as solid after removing the solvent inside the glove box. 

**Conversion of (1-octyl)_2_Zn to (1-octyl)Li.** (1-Octyl)_2_Zn (58.4 mg, 0.200 mmol) was added to a solution of *t*-BuLi (25.6 mg, 0.400 mmol) in methylcyclohexane (27.0 g). After stirring for 15 min at room temperature, volatiles were removed using a vacuum line. A light-yellow oil was obtained, of which the ^1^H and ^13^C NMR spectra agreed with those of 1-octyllithium. Differently, (1-octyl)_2_Zn (0.29 g, 1.0 mmol) was added to a solution of *t*-BuLi (0.13 g, 2.0 mmol) in decane (10 g). The solution was stirred for 20 min at 130 °C while venting off the generated gases. A black solid was generated, which was filtered through Celite. Decane was distilled at 50 °C under full vacuum to obtain a light-yellow oil of which the ^1^H and ^13^C NMR spectra agreed with those of 1-octyllithium (0.22 g, 91%). ^1^H NMR (C_6_D_6_): δ 1.54 (s, 2H, CH_2_), 1.49-1.30 (br, 10H, CH_2_), 0.94 (t, *J* = 7.2 Hz, 3H, CH_3_), 0.33 (s, 2H, LiCH_2_) ppm. ^13^C NMR (C_6_D_6_): δ 38.79, 32.50, 32.23, 29.94, 29.79, 29.68, 23.20, 14.43 ppm.

**1-Octene, *n*-BuLi, and PMDTA in methylcyclohexane.***n*-BuLi (1.10 g, 17.3 mmol) was added dropwise to a solution containing PMDTA (3.00 g, 17.3 mmol) and 1-octene (3.90 g, 34.6 mmol) in methylcyclohexane (77 g). After stirring overnight at room temperature, the yellowish solution (2.16 mmol-Li/g) was used for the styrene polymerizations. 

**Me_2_NCH_2_CH_2_N(Me)CH_2_CH_2_N(Me)CH_2_Li.***sec*-BuLi (12.8 mg, 0.200 mmol) was added dropwise to a solution of PMDTA (34.6 mg, 0.200 mmol) in methylcyclohexane (1.50 g). After stirring for 30 min at room temperature, the solution (0.129 mmol-Li/g) was used for the styrene polymerizations. Additionally, *sec*-BuLi (12.8 mg, 0.200 mmol) and PMDTA (34.6 mg, 0.200 mmol) were dissolved in C_6_D_12_ (~0.5 mL) and an ^1^H NMR spectrum was recorded after 30 min.

**Me_2_NCH_2_CH_2_N(Me)Li.***n*-BuLi (10 mL, 1.65 M, 16.5 mmol) was added dropwise to a solution of Me_2_NCH_2_CH_2_N(Me)H (1.69 g, 16.5 mmol) in hexane (25 mL). After stirring for 5 h at room temperature, the resulting solution was filtered through Celite. The solvent was removed using a vacuum line, and we obtained a white solid (1.56 g, 88%) that was used for styrene polymerizations after adding it to an equivalent amount of PMDTA in methylcyclohexane. ^1^H NMR (C_6_D_6_): δ 3.21 (br, 2H, CH_2_), 3.11 (br, 3H, NLi(CH_3_)), 2.45 (br, 2H, CH_2_), 1.98 (br, 6H, N(CH_3_)_2_) ppm. 

**Pentylallyl-Li⋅(PMDTA).***n*-BuLi (0.14 mg, 2.2 mmol) was added dropwise to PMDTA (0.37 g, 2.2 mmol) in 1-octene (13.0 g). After stirring overnight at room temperature, the yellowish solution (0.16 mmol-Li/g) was used for the styrene polymerizations. An aliquot was analyzed with ^1^H NMR spectroscopy. After recording the ^1^H NMR spectrum, the solution in C_6_D_6_ was quenched with H_2_O (or D_2_O) and filtered over a short pad of anhydrous MgSO_4_ in a pipette to re-record an ^1^H NMR spectrum.

**PhLi⋅(PMDTA).***n*-BuLi (12.8 mg, 0.200 mmol) was added dropwise to a solution of PMDTA (34.6 mg, 0.200 mmol) in C_6_D_6_ (0.600 g). After stirring for 30 min at room temperature, the solution (0.31 mmol-Li/g) was analyzed with ^1^H NMR spectroscopy and used for the styrene polymerizations. 

**PS chain growth from (1-hexyl)_2_Zn.** Pentylallyl-Li⋅(PMDTA) (96 µmol) was added to a flask containing (1-hexyl)_2_Zn (22.6 mg, 96 µmol) and methylcyclohexane (27 g) inside a glove box. Styrene (5.0 g, 48.0 mmol) was added, and the anionic polymerization was performed at 90 °C for 5 h. Then, aqueous HCl (2 N, 0.3 mL) was added, and the resulting solution was stirred for 30 min at 90 °C to destroy the zinc species. The solution was filtered through a short pad of silica gel, which was subsequently washed with toluene. In order to isolate PS, toluene was removed with a rotary evaporator; the isolated sample was dried in a vacuum oven at 130 °C for 5 h (5.00 g, 100%). 

**Synthesis of poly(ethylene-*co*-propylene)-*b*-PS.** A bomb reactor (125 mL) was evacuated at 60 °C for 1 h. After filling the reactor with ethylene gas at atmospheric pressure, a solution of Me_3_Al (29.0 mg, 200 µmol-Al) in methylcyclohexane (15.5 g) was added. The mixture was stirred for 40 min at 100 °C using a mantle, and the solution was subsequently removed using a cannula. The reactor was evacuated again to remove any residual solvent, and it was filled with ethylene/propylene gas at atmospheric pressure. This washing procedure was performed to remove any catalyst poisons. The reactor was charged with methylcyclohexane (15.5 g) containing MMAO (modified-methylaluminoxane, AkzoNobel, 6.7 wt%-Al in heptane, 20 mg, 50 µmol-Al) and the temperature was set to 80 °C. A solution of (1-hexyl)_2_Zn (35.4 mg, 150 µmol-Zn) in methylcyclohexane (10.0 g) and a solution of **1** in cyclohexane (8.7 μmol/g, 230 mg, 2.0 µmol) diluted with methylcyclohexane (0.5 g) were successively injected. An ethylene/propylene mixed gas (10 bar/15 bar, total 25 bar) was charged from a tank into the reactor at 25 bar, and the mixture was polymerized for 40 min. The temperature increased spontaneously to 110 °C within 5 min and was subsequently maintained at 90–100 °C with a controller. The pressure in the tank decreased from 23 to 21 bar. After the remaining ethylene/propylene mixed gas was vented off, an aliquot was taken for a GPC study. Pentylallyl-Li⋅(PMDTA) (200 µmol) in methylcyclohexane (10.0 g) was injected at 95 °C. After stirring for 15 min at 95 °C, a solution of styrene (5.0 g) in methylcyclohexane (5.0 g) was injected, and the mixture was polymerized for 4 h while controlling the temperature within the range of 90–100 °C. An aliquot was taken for ^1^H NMR spectroscopy; the spectrum showed no signals due to the styrene monomer. Acetic acid (2.0 mL) and ethanol (30 mL) were successively injected into the reactor. The generated polymer was dried in a vacuum oven at 160 °C (18.1 g). After dissolving the polymer (3.0 g) in chloroform (30 g) at 60 °C for 3 h, acetone (60 g) was added to precipitate the PO-*block*-PS. Homo-PS, which is soluble in chloroform/acetone mixed solvents, was isolated by filtration.

## 3. Results and Discussion

### 3.1. Converting Dialkylzinc to Alkyllithium 

**Scheme 2:**
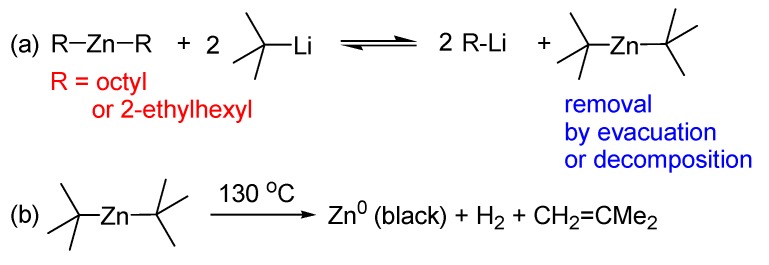
Converting R_2_Zn to RLi.

**Figure 1:**
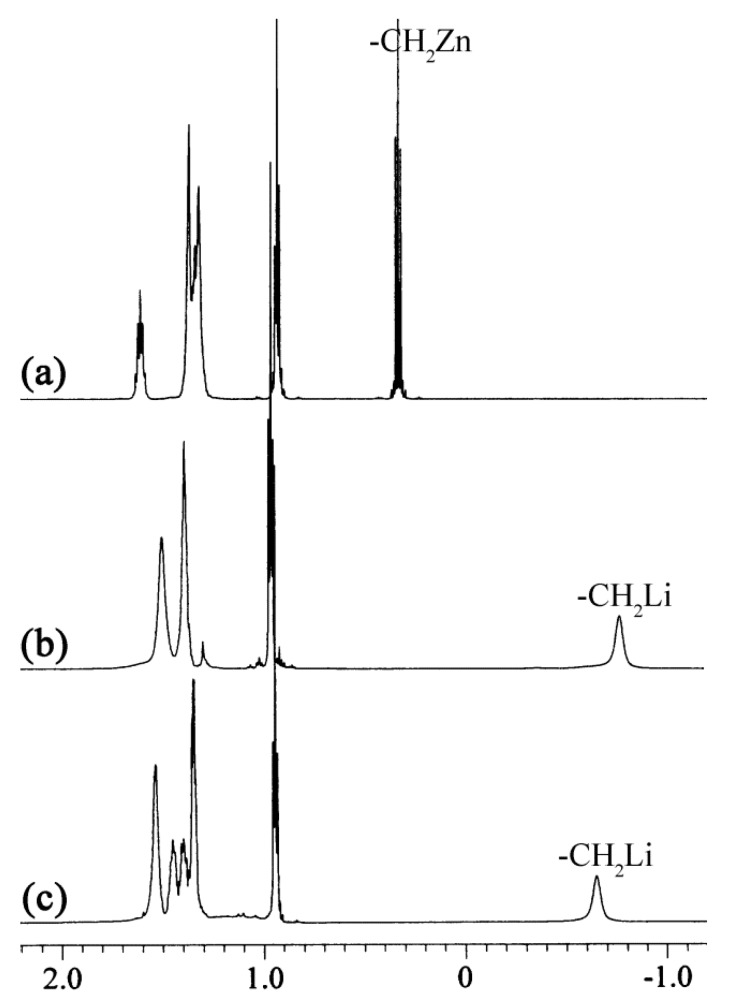
^1^H NMR spectra of: (**a**) (1-Octyl)_2_Zn; (**b**) Commercial source of 1-hexyllithium; (**c**) 1-Octyllithium generated from (1-octyl)_2_Zn.

Alkyllithium is a very reactive species commonly used in living anionic polymerizations as an initiator. A developed method to convert dialkylzinc to alkyllithium would be a powerful tool for the syntheses of various types of PO-based block copolymers (Scheme 1) [39]. Alkyllithium is a more reactive species than the corresponding dialkylzinc, and the reaction for converting alkyllithium to dialkylzinc is conventionally adopted, whereas its reverse reaction, i.e., converting dialkylzinc to alkyllithium is not favored and not yet realized. We expected that the addition of very reactive and bulky *t*-BuLi (2.0 eq) to dialkylzinc (e.g., (1-octyl)_2_Zn) might transiently generate 1-octyllithium and (*t*Bu)_2_Zn and that the generated (*t*Bu)_2_Zn might be selectively removed from the reaction pot through evacuation or decomposition at a high temperature (Scheme 2). The removal of volatiles under full vacuum from a flask containing (1-octyl)_2_Zn and *t*-BuLi in methylcyclohexane yielded 1-octyllithium in 91% yield. In the ^1^H NMR spectrum of the remaining, we observed a set of signals that was assigned to 1-octyllithium, especially by comparison with the signals of the commercial source of 1-hexyllithium (Figure 1, and Figure S1 for ^13^C NMR spectrum). The use of bulky *t*-BuLi was essential for the successful conversion of (1-octyl)_2_Zn to 1-octyllithium; when *n*-BuLi, *sec*-BuLi, or Me_3_SiCH_2_Li were used instead of *t*-BuLi, only a mixture of alkyllithium and (1-octyl)_2_Zn remained after evacuation, and not 1-octyllithium.

The transiently generated (*t*Bu)_2_Zn could also be removed via selective decomposition at a high temperature of 130 °C. (Primary alkyl)_2_Zn compounds (e.g., Et_2_Zn and (1-octyl)_2_Zn) are stable up to 150 °C and can be readily used in CCTP as CTAs at high temperatures of 125–140 °C [23]. In contrast, we found that (*t*Bu)_2_Zn was decomposed at 130 °C, and a black solid precipitated when a solution of (*t*Bu)_2_Zn in decane was heated at 130 °C. Isobutene and H_2_ signals were detected in the ^1^H NMR spectrum when the reaction was performed in a sealed tube in toluene-d_8_ (Scheme 2b). (Primary alkyl)lithium, e.g., *n*-BuLi, was negligibly decomposed in decane at 130 °C (half-life, 6 h) [40]. We found that *t*-BuLi was also persistent at 130 °C for a short time of ~30 min. Accordingly, when a solution of (1-octyl)_2_Zn and *t*-BuLi (2.0 eq) in decane was heated at 130 °C for 30 min, a black solid precipitated, which was indicative of the decomposition of (*t*Bu)_2_Zn; 1-octyllithium was cleanly isolated from the reaction pot by filtration in 91% yield. When benzaldehyde was added after the thermal treatment, PhCH(OH)(CH_2_)_7_CH_3_ was afforded, which additionally supported the successful generation of 1-octyllithium; (1-octyl)_2_Zn does not react with benzaldehyde. Employing the same method, (2-ethylhexyl)_2_Zn was also converted to 2-ethylhexyl-Li in high yield (84%; Figure S2).

### 3.2. Attempts to Synthesize Block Copolymers

(Polyolefinyl)_2_Zn was prepared via coordinative chain transfer copolymerizations (CCTcoPs) performed using a pyridylamidohafnium catalyst (**1** in Scheme 1) in methylcyclohexane at high temperatures of 90–110 °C by feeding ethylene/propylene mixed gases. Catalyst **1** is the best in performing CCTcoPs [22,33,41,42,43]. It undergoes fast alkyl exchange with Zn sites to generate PO chains with a narrow molecular weight distribution [21,44]. The β-elimination process can be avoided with **1**, preventing the generation of PO chains that are not attached to Zn sites [18,45,46]. Moreover, **1** is capable of incorporating a significant amount of α-olefins in an ethylene/α-olefin copolymerization [47]. A minimal amount of MMAO (50 μmol-Al) had to be fed, in addition to the CTA (1-hexyl)_2_Zn (100 or 200 μmol-Al), to realize the full activity of **1**, even though PO chain growth from some Al-sites was unavoidable [26,48,49]. The generated (polyolefinyl)_2_Zn was treated with *t*-BuLi ([Li] = 2 × [Zn] + [Al], i.e., 250 or 450 μmol) at 130–135 °C for 1.0 h aiming to generate polyolefinyl-Li by destroying the transiently generated (*t*Bu)_2_Zn. Among other monomers, styrene (5.0 g) was fed, aiming to grow a PS chain initiating from polyolefinyl-Li. All the fed styrene monomers were completely converted to polymer in 4 h, but the desired PO-*block*-PS was not generated. In GPC studies, two signals were observed in opposite directions relative to the base line: A very high molecular weight negative signal (*M*_n_ 1 150 000, *M*_w_/*M*_n_ 1.2) assigned to homo-PS, and a main positive signal assigned to PO; the *M*_n_ value of this signal (*M*_n_ 61 000, *M*_w_/*M*_n_ 2.3) was not increased relative to that of the homo-PO sample taken before feeding styrene (*M*_n_ 65 000, *M*_w_/*M*_n_ 2.1, Figure S3, entry 1 in Table 1). This observation indicated that the isolated polymer was not a block copolymer but a mixture of homo-PO and homo-PS. However, when PMDTA was added alongside the styrene monomer, the high molecular weight homo-PS signal disappeared, and unimodal curves were observed with narrow molecular weight distributions (*M*_w_/*M*_n_ l.3–1.5). Moreover, after the styrene polymerization, the GPC curves were shifted to a high molecular weight direction with a significant increase in the *M*_n_ values (Δ*M*_n_ 13–41 kDa), indicating the generation of the desired PO-*block*-PS (Figure S4, entries 2–5).

Attempts to grow other polymer chains (e.g., polyisoprene and polycaprolactone) initiating from polyolefinyl-Li, which was assumed to be generated, were unsuccessful. After performing anion polymerization of isoprene, the GPC curves were not shifted to a high molecular weight direction, i.e., the *M*_n_ values increased negligibly. We eventually found that *t*-BuLi reacted with olefin monomers; hence, we attempted to convert the CCTP product (polyolefinyl)_2_Zn to polyolefinyl-Li by thoroughly flushing ethylene/propylene residual gases before adding *t*-BuLi. However, many attempts were also unsuccessful. Either the low concentration of Zn species relative to that in the (1-octyl)_2_Zn model studies or the difficulty of the formation of aggregates, in the case of polyolefinyl-Li, might have caused the failure in converting (polyolefinyl)_2_Zn to polyolefinyl-Li.

### 3.3. Initiators for PS Chain Growth from Dialkylzinc

**Scheme 3:**
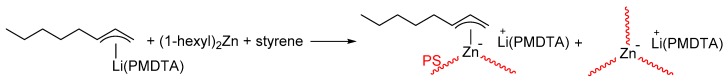
PS chain-growth process from dialkylzinc.

PO-*block*-PSs were efficiently generated when the CCTP product (polyolefinyl)_2_Zn was treated with *t*-BuLi and PMDTA in the presence of residual propylene gas. We assumed that allyl-Li⋅(PMDTA), generated from the reaction of *t*-BuLi, propylene and PMDTA, might work as an efficient initiator to grow PS chains from (polyolefinyl)_2_Zn (Scheme 3). Therefore, we prepared a reaction of 1-octene with *n*-BuLi in methylcyclohexane in the presence of PMDTA. After overnight stirring, *n*-BuLi signals completely disappeared from the ^1^H NMR spectrum, and the resulting solution was used as an initiator for styrene polymerization in the presence of (1-hexyl)_2_Zn (entries 1–3 in Table 2). The number of PS chains was calculated by dividing the isolated PS weight by the measured *M*_n_ value. The obtained PS chains were twofold the fed Zn amount (205, 203, and 203 μmol vs. 2 × 100 = 200 μmol), and their numbers were unaltered by the amount of the fed lithium species (50, 70, and 100 μmol, respectively). These observations indicated that the PS chains were grown selectively from all the fed (1-hexyl)_2_Zn and that the lithium species worked only as an activator in the PS chain-growth process, not directly engaging as a PS chain-growing site. One disadvantage was that the styrene monomers were not completely converted to polymer, affording a 92–96% yield, even considering the long reaction time of 5 h at a high temperature of 90 °C. The molecular weight distributions were rather broad (*M*_w_/*M*_n_, 1.35–1.45).

We observed broad and unassignable signals in the ^1^H NMR spectrum of the lithium species generated in the reaction pot of “1-octene + *n*-BuLi + PMDTA” in methylcylohexane (Figure S5). However, the spectra recorded after quenching with H_2_O and D_2_O, indicated the presence of pentylallyl-Li, Me_2_NCH_2_CH_2_N(Me)Li, and Me_2_NCH_2_CH_2_N(Me)CH_2_CH_2_N(Me)CH_2_Li (Figure S6). The reaction of *n*-BuLi with PMDTA in C_6_D_12_ was monitored with ^1^H NMR spectroscopy, which revealed that *n*-BuLi reacted with PMDTA slowly, requiring ~8 h at room temperature for the complete consumption of *n*-BuLi, to generate mainly Me_2_NCH_2_CH_2_N(Me)CH_2_CH_2_N(Me)CH_2_Li (Figure S7) [50,51]. The generated Me_2_NCH_2_CH_2_N(Me)CH_2_CH_2_N(Me)CH_2_Li was unstable; thus, it converted to Me_2_NCH_2_CH_2_N(Me)Li, Me_2_NLi, and PMDTA [45]. *sec*-BuLi reacted with PMDTA within 30 min at room temperature, to generate mainly Me_2_NCH_2_CH_2_N(Me)CH_2_CH_2_N(Me)CH_2_Li in C_6_D_12_ (Figure S8) [52]. PMDTA treated with *n*-BuLi in C_6_D_6_ cleanly afforded C_6_D_5_Li⋅(PMDTA) (Figure S9). When PMDTA was mixed with *n*-BuLi in 1-octene (as a solvent as well as a reactant), the color of the solution slowly turned to yellow. The ^1^H NMR spectrum of the lithium species generated in the reaction pot of “PMDTA + *n*-BuLi” in 1-octene was ambiguous; however, the signals assigned to 2-octene (as a mixture of cis- and trans-isomers) and 1-octene were observed after quenching with H_2_O, indicating the generation of pentylallyl-Li species in the reaction pot of “PMDTA + *n*-BuLi” in 1-octene (Figure S10).

Upon these observations, organolithium species that might be generated by the reaction of BuLi, olefin, and PMDTA, were screened as initiators for styrene polymerization in the presence of (1-hexyl)_2_Zn (Table 2). Organolithium species generated in situ or prepared were fed in the polymerization pot containing styrene (5.0 g) and (1-hexyl)_2_Zn (100 μmol) in methylcyclohexane and polymerization was performed at 90 °C for 5 h. The numbers of PS chain-growing sites were calculated by dividing the isolated PS weights by the measured *M*_n_ values, which were monitored to see whether PS chains were well-grown from (1-hexyl)_2_Zn. When Me_2_NCH_2_CH_2_N(Me)CH_2_CH_2_N(Me)CH_2_Li (100 μmol) was generated, in situ, in the reaction pot of “*sec*-BuLi + PMDTA in methylcyclohexane” in 30 min was used (entry 4), styrene monomers were not completely converted to PS (95% yield) and the calculated number of PS chain-growing sites was 240 μmol, exceeding the value of “2 × Zn (μmol)”, but not surpassing the value of “2 × Zn (μmol) + Li (μmol)”. The molecular weight distribution was narrow (*M*_w_/*M*_n_, 1.25). When Me_2_NCH_2_CH_2_N(Me)Li was used (entry 5), styrene conversion was unsatisfactorily low (23%). However, when Me_2_NCH_2_CH_2_N(Me)Li·(PMDTA) was used instead (entries 6–8), the conversions were high but not quantitative (91–93%). The calculated number of PS chains agreed well with the value of “2 × Zn (μmol)” (217, 208, and 195 μmol versus 2 × 100 μmol) and it was almost unaffected by the increase in the feed amount of lithium species (50, 70, and 100 μmol, respectively), which indicated that the PS chains were grown selectively from all the fed Zn species, and not from Me_2_NCH_2_CH_2_N(Me)Li. 

When pentylallyl-Li⋅(PMDTA) generated in situ in the reaction pot of “*n*-BuLi + PMDTA” in 1-octene was used (entries 9–11), styrene monomers were completely converted to PS, and the number of PS chain-growing sites exceeded the value of “2 × Zn (μmol)” (233, 240, and 258 μmol, respectively) and it increased with the increase in the feed amount of lithium species (50, 70, and 100 μmol, respectively). These observations indicated that PS chains were grown from all the Zn sites, as well as from some portion of the fed organolithium species (Scheme 3). PhLi⋅(PMDTA) showed similar results with pentylallyl-Li, which exhibited performance comparable to that of Me_3_SiCH_2_Li⋅(PMDTA) and *n*-BuLi⋅(PMDTA)—previously introduced as initiators in growing PS chains from dialkylzinc (entries 15 and 16) [33,45]. Styrene monomers were quantitatively converted to PS and the numbers of PS chains were comparable, exceeding the value of “2 × Zn (μmol)” (220–260 μmol), in all cases. The molecular weight distributions in the cases of pentylallyl-Li⋅(PMDTA), PhLi⋅(PMDTA), and Me_3_SiCH_2_Li⋅(PMDTA) were narrow (*M*_w_/*M*_n_ 1.24–1.30), while the distribution in the case of *n*-BuLi⋅(PMDTA) was rather broad (*M*_w_/*M*_n_ 1.48).

### 3.4. Synthesis of PO-Block-PS 

CCTcoPs were performed with (1-hexyl)_2_Zn (150 or 300 μmol) as CTA using **1** as a catalyst by feeding an ethylene/propylene mixed gas to generate (polyolefinyl)_2_Zn. After CCTcoP, lithium species ([Li] = [Zn] + [Al], i.e., 450 or 650 μmol) and styrene monomers (5.0 or 10 g) were sequentially fed and styrene polymerization was performed for 4 h, at a reasonably high temperature of 90–100 °C to prevent precipitation of the generated polymers. Running the styrene polymerization at higher temperature up to 120 °C was not problematic. At the initial stage of the styrene polymerization, a clear yellowish solution was developed, which became turbid for approximately 5 min, and eventually turned back to a clear yellowish viscous solution once the block copolymers were well-generated. When the block copolymers were not generated well (e.g., entry 1 in Table 1 and entry 3 in Table 3), the polymerization solution was turbid throughout the styrene polymerization. The isolated PO-*block*-PS polymers were transparent, while mixtures of homo-PO and homo-PS were opaquely white. 

When the lithium species generated in the reaction pot of “1-octene + *n*-BuLi + PMDTA” in methylcyclohexane was used as an initiator, the styrene monomers were completely converted to polymer. However, the increase in the molecular weight was marginal after the styrene polymerization (Δ*M*_n_ 5 kDa, entry 1 in Table 3). The molecular weight distribution was also marginally narrowed from an *M*_w_/*M*_n_ value of 1.75 to 1.64 after the styrene polymerization. When Me_2_NCH_2_CH_2_N(Me)CH_2_CH_2_N(Me)CH_2_Li generated in the reaction pot of “*sec*-BuLi + PMDTA” in methylcyclohexane was used, the styrene polymerization was not initiated at all. When Me_2_NCH_2_CH_2_N(Me)Li·(PMDTA) was used, the styrene monomers were partially converted to PS (~60% conversion, entry 3). 

**Figure 2:**
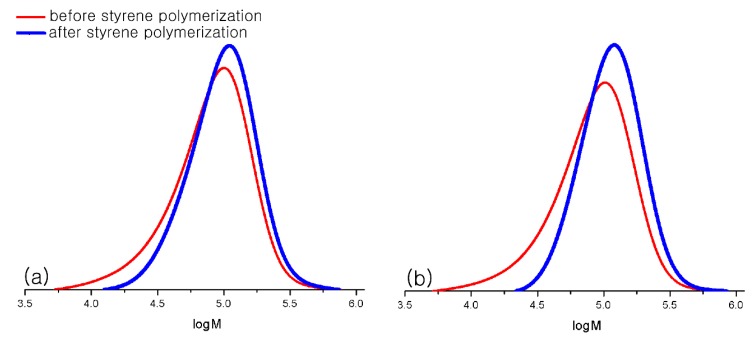
GPC curves of the polymers before and after styrene polymerization: (**a**) entry 4; (**b**) entry 5 in Table 2.

Pentylallyl-Li⋅(PMDTA) generated in the reaction pot of “*n*-BuLi + PMDTA” in 1-octene was the best initiator. The GPC curves were shifted to a high molecular weight direction after the styrene polymerization (Figure 2 and Figure S11). Increases in the *M*_n_ values, after styrene polymerization, were substantial and reasonable (Δ*M*_n_ 22, 37, 11, 20 kDa, entries 4–7). By feeding the amount of styrene monomers twice under otherwise identical conditions, the Δ*M*_n_ values almost doubled from 22 kDa to 37 kDa and from 11 kDa to 20 kDa. By feeding twice the amount of Zn species in CCTcoP and accordingly twice the amount of lithium species under otherwise identical conditions, the Δ*M*_n_ values were reduced almost by half from 22 kDa to 11 kDa and from 37 kDa to 20 kDa. The molecular weight distributions were also substantially narrowed after the styrene polymerization from the *M*_w_/*M*_n_ values of 1.61, 1.61, 1.50, and 1.54, to 1.39, 1.30, 1.35, and 1.26, respectively (ΔPDI 0.22, 0.31, 0.15, and 0.28). A weak melting (*T*_m_) signal corresponding to poly(ethylene-*co*-propylene) block and a glass transition (*T*_g_) signal corresponding to PS block were independently observed at a broad range of 30–80 °C and ~100 °C, respectively, on differential scanning calorimetry (DSC) (Figure S12).

**Scheme 4:**
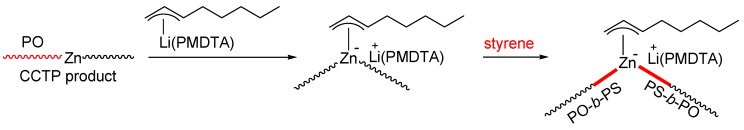
Synthetic scheme for PO-*block*-PS.

Homo-PS could be separated from the block copolymers by extraction with an acetone/chloroform mixed solvent. The extracted homo-PS was ~1/3 (27–29%) of the amount of the total consumed styrene, from which we hypothesized that the PS chains were grown from both polyolefinyl and pentylallyl groups in the zincate species [(polyolefinyl)_2_(pentylallyl)Zn]^−^[Li⋅(PMDTA)]^+^ formed by the action of pentylallyl-Li⋅(PMDTA) onto (polyolefinyl)_2_Zn; PS chain growth from polyolefinyl groups results in the generation of the desired poly(ethylene-*co*-propylene)-*block*-PS, while that from pentylallyl generates homo-PS in 1/3 of the total consumed styrene. However, the number of the PS chain-growing sites calculated by dividing the weights of the total consumed styrene by the measured homo-PS *M*_n_ values, did not match the value of “3 × Zn (μmol)”, opposing the hypothesis. Conversely, that number agreed with the value of “2 × Zn (μmol)” (310 and 360 μmol vs. 2 × 150 = 300 μmol for entries 4 and 5; 630 μmol vs. 2 × 300 = 600 μmol for entry 7). Thus, we hypothesized that the PS chains were grown mainly from polyolefinyl groups in the formed zincate species [(polyolefinyl)_2_(pentylallyl)Zn]^−^[Li⋅(PMDTA)]^+^ (Scheme 4); we attributed the extracted homo-PS to the PS chains grown either from the 1-hexyl group, which may remain intact during CCTP or from the polyolefinyl groups, which are grown shortly in CCTP. When the feed amount of Zn species was high (300 μmol) and the feed amount of styrene monomers was too low (5.0 g) (entry 6), the number of PS chain-growing sites did not exceed the value of “2 × Zn (μmol)” (450 μmol vs. 2 × 300 = 600 μmol), which indicated that the PS chains were not grown from all the polyolefinyl-Zn groups. The molecular weight distributions of the extracted homo-PS were fairly narrow (*M*_w_/*M*_n_ 1.23–1.25), indicating that the anionic styrene polymerization was well-controlled. 

PhLi⋅(PMDTA) was as effective an initiator as pentylallyl-Li⋅(PMDTA) for growing PS chains from (polyolefinyl)_2_Zn. Increases of the *M*_n_ values after the styrene polymerization were substantial (Δ*M*_n_ 12, 38, 10, 21 kDa, entries 8–11; Figure S11), and the molecular weight distributions were significantly narrowed after the styrene polymerization, with the *M*_w_/*M*_n_ values going from 1.65, 1.63, 1.58, and 1.64 to 1.49, 1.29, 1.41, and 1.34, respectively. However, the amounts of the extracted homo-PSs were slightly higher (30–34% vs. 27–29%) and the molecular weight distributions of the extracted homo-PSs were broader than those in the case of pentylallyl-Li⋅(PMDTA) (*M*_w_/*M*_n_ 1.38–1.52 vs. 1.23–1.25), indicating that pentylallyl-Li⋅(PMDTA) might be a better initiator than PhLi⋅(PMDTA) in growing PS chains from (polyolefinyl)_2_Zn. Previously, we introduced *n*-BuLi⋅(TMEDA) and Me_3_SiCH_2_Li⋅(PMDTA) as initiators for growing PS chains from the CCTP product (polyolefinyl)_2_Zn, mainly based on the model studies performed with R_2_Zn (R = Et, 1-hexyl, benzyl) [32,33]. Though significant performance differences could not be observed among pentylallyl-Li⋅(PMDTA), PhLi⋅(PMDTA), n-BuLi⋅(TMEDA), and Me_3_SiCH_2_Li⋅(PMDTA) in the model studies performed with (1-hexyl)_2_Zn (Table 2), the studies performed with actual (polyolefinyl)_2_Zn indicated that pentylallyl-Li⋅(PMDTA) and PhLi⋅(PMDTA) were superior to the previous initiators. When *n*-BuLi⋅(PMDTA) was used, a substantial amount of homo-PS was generated (45%, entry 12). When Me_3_SiCH_2_Li⋅(PMDTA) was used, the increase in the *M*_n_ value after performing the styrene polymerization was not as substantial as that observed for pentylallyl-Li⋅(PMDTA) or PhLi⋅(PMDTA) (entry 13).

## 4. Conclusions

Developing a versatile synthetic tool for polyolefin-base block copolymers, we unsuccessfully attempted to convert the CCTP product (polyolefinyl)_2_Zn to polyolefinyl-Li though 1-octyl-Li was efficiently synthesized from (1-octyl)_2_Zn (a model compound of (polyolefinyl)_2_Zn). However, an efficient initiator to grow PS chains from (polyolefinyl)_2_Zn was eventually found. Pentylallyl-Li⋅(PMDTA) (generated in a pot containing *n*-BuLi and PMDTA in 1-octene) and styrene monomers were added to a reactor containing (polyolefinyl)_2_Zn generated via CCTcoP, affording the desired poly(ethylene-*co*-propylene)-*block*-PSs. The M_n_ values increased substantially after the styrene polymerization, and the increments (i.e., Δ*M*_n_ values) were reasonable. By feeding twice the amount of styrene, the increments doubled and, by feeding twice the amount of Zn species in CCTcoP, and accordingly, twice the amount of lithium species in styrene polymerization, the increments were reduced by half. Homo-PS was concomitantly generated at 27–29% the amount of the total consumed styrene. The numbers of PS chain-growing sites were calculated by dividing the weights of the total consumed styrene with the measured homo-PS *M*_n_ values, which roughly agreed with the value of “2 × Zn (μmol)”, indicating the growth of PS chains mainly from polyolefinyl groups in zincate species [(polyolefinyl)_2_(pentylallyl)Zn]^−^[Li⋅(PMDTA)]^+^ formed by the action of pentylallyl-Li⋅(PMDTA) onto (polyolefinyl)_2_Zn. Pentylallyl-Li⋅(PMDTA) was superior to *n*-BuLi⋅(PMDTA) and Me_3_SiCH_2_Li⋅(PMDTA)—previously introduced as initiators to grow PS chains from (polyolefinyl)_2_Zn. Thus, pentylallyl-Li⋅(PMDTA) may be useful in the production of the commercially relevant PS-*block*-PO-*block*-PS copolymer [33,34,52].

## Figures and Tables

**Table 1:** Results for preparation of poly(ethylene-*co*-propylene)-*b*-PS ^a^.

Entry	(1-hexyl)_2_Zn (μmol)	*t*-BuLi (μmol)	PO (g); *F*_C3_ (mol%) ^b^	PS (g); Homo Fraction (%)	*M*_n_ (kDa); PDI before Styrene Polym ^c^	*M*_n_ (kDa); PDI after Styrene Polym ^c^
1 ^d^	100	250	13.1; 23.4	5.0; -	64.6 (2.10)	61.3 (2.30)
2	100	250	11.4; 20.5	5.0; 21	108 (1.48)	121 (1.48)
3	100	250	12.5; 22.6	10; 27	92 (1.62)	111 (1.54)
4	200	450	12.9; 22.4	5.0; 28	51 (1.66)	75 (1.33)
5	200	450	15.2; 22.8	10; 30	48 (1.74)	89 (1.28)

^a^ Polymerization conditions: methylcyclohexane (26 g), catalyst (2.0 μmol), and MMAO (modified-methylaluminoxane) as a scavenger (50 μmol-Al) for coordinative chain transfer polymerization (CCTP) and then t-BuLi and styrene (5 or 10 g) in methylcyclohexane (15 g) and pentamethyldiethylenetriamine (PMDTA) ([PMDTA] = [Li]) for anionic polymerization. ^b^ Propylene content in poly(ethylene-*co*-propylene) block was calculated from ^1^H NMR spectra. ^c^ Measured with gel permeation chromatography (GPC) at 160 °C using trichlorobenzene relative to polystyrene (PS) standards. ^d^ Styrene polymerization was performed in the absence of PMDTA.

**Table 2:** Results of anionic styrene polymerization in the presence of (1-hexyl)_2_Zn (100 μmol) ^a^.

Entry	Initiator	Li (μmol)	yield (g; %)	*M*_n_ (Da) ^b^	*M*_w_/*M*_n_	# of PS chains (μmol) ^c^
1	1-octene + *n*-BuLi + PMDTA in MeCy	50	4.69; 94	22,900	1.45	205
2	1-octene + *n*-BuLi + PMDTA in MeCy	70	4.62; 92	22,800	1.39	203
3	1-octene + *n*-BuLi + PMDTA in MeCy	100	4.82; 96	23,800	1.35	203
4	Me_2_NCH_2_CH_2_N(Me)CH_2_CH_2_N(Me)CH_2_Li	100	4.75; 95	19,700	1.25	240
5	Me_2_NCH_2_CH_2_N(Me)Li	100	1.14; 23	7400	2.10	154
6	Me_2_NCH_2_CH_2_N(Me)Li·(PMDTA)	50	4.56; 91	21,000	1.32	217
7	Me_2_NCH_2_CH_2_N(Me)Li·(PMDTA)	70	4.63; 93	22,300	1.33	208
8	Me_2_NCH_2_CH_2_N(Me)Li·(PMDTA)	100	4.67; 93	24,000	1.27	195
9	pentylallyl-Li⋅(PMDTA)	50	5.00; 100	21,500	1.28	233
10	pentylallyl-Li⋅(PMDTA)	70	5.00; 100	20,800	1.24	240
11	pentylallyl-Li⋅(PMDTA)	100	5.00; 100	19,400	1.30	258
12	PhLi⋅(PMDTA)	50	5.00;100	22,000	1.30	227
13	PhLi⋅(PMDTA)	70	4.98; 99	21,100	1.27	236
14	PhLi⋅(PMDTA)	100	4.98; 99	21,000	1.24	237
15	*n*-BuLi⋅(PMDTA)	100	4.96; 99	21,000	1.48	236
16	Me_3_SiCH_2_Li⋅(PMDTA)	100	5.00; 100	23,000	1.25	217

^a^ Polymerization conditions: (1-hexyl)_2_Zn (22.6 mg, 100 μmol), methylcyclohexane (27 g), styrene (5.0 g, 48 mmol), 90 °C, 5 h. ^b^ Measured by GPC at 40 °C using toluene eluent. ^c^ Calculated as yield (g)/*M*_n_.

**Table 3:** Results for preparation of poly(ethylene-*co*-propylene)-*b*-PS ^a^.

Entry	(hexyl)_2_Zn (μmol)	Initiator	PO (g); *F*_C3_ (mol%) ^b^	PS (g); Homo Fraction (%)	Homo-PS *M*_n_ (kDa); PDI	*M*_n_ (kDa); PDI before Styrene Polym ^c^	*M*_n_ (kDa); PDI after Styrene Polym*^c^*
1	150	1-octene + *n*-BuLi + PMDTA in MeCy	15.6; 22	5.0; 29	24 (1.41)	61 (1.75)	66 (1.64)
2	150	Me_2_NCH_2_CH_2_N(Me)CH_2_CH_2_N(Me)CH_2_Li	12.4; 21	~0	0	60 (1.65)	59 (1.65)
3	150	Me_2_NCH_2_CH_2_N(Me)Li·(PMDTA)	15.9; 23	3.5; 30	39 (2.77)	60 (1.76)	64 (1.70)
4	150	pentylallyl-Li⋅(PMDTA)	13.1; 17	5.0; 29	16 (1.25)	60 (1.61)	82 (1.39)
5	150	pentylallyl-Li⋅(PMDTA)	13.5; 21	10; 28	27 (1.24)	62 (1.61)	99 (1.30)
6	300	pentylallyl-Li⋅(PMDTA)	14.2; 22	5.0; 27	11 (1.23)	40 (1.50)	51 (1.35)
7	300	pentylallyl-Li⋅(PMDTA)	13.0; 19	10; 28	16 (1.24)	35 (1.54)	54 (1.26)
8	150	PhLi⋅(PMDTA)	15.2; 24	5.0; 30	20 (1.52)	64 (1.65)	76 (1.49)
9	150	PhLi⋅(PMDTA)	13.0; 17	10; 34	28 (1.39)	67 (1.63)	105 (1.29)
10	300	PhLi⋅(PMDTA)	12.0; 22	5.0; 30	11 (1.40)	33 (1.58)	43 (1.41)
11	300	PhLi⋅(PMDTA)	14.8; 23	10; 33	16 (1.38)	38 (1.64)	59 (1.34)
12	150	*n*-BuLi⋅(PMDTA)	14.6; 24	5.0; 45	23 (1.33)	63 (1.73)	71 (1.65)
13	150	Me_3_SiCH_2_Li⋅(PMDTA)	16.0; 21	5.0; 27	19 (1.35)	71 (1.59)	76 (1.49)

^a^ Polymerization conditions: methylcyclohexane (26 g), catalyst (2.0 μmol), and MMAO (50 μmol-Al) as a scavenger for CCTP and then lithium compound ([Li] = [Zn] + [Al]) and styrene (5.0 g or 10 g) in methylcyclohexane (15 g) for anionic polymerization. ^b^ Propylene content in poly(ethylene-*co*-propylene) block was calculated from ^1^H NMR spectra. ^c^ Measured by GPC at 160 °C using trichlorobenzene relative to PS standards.

## Supplementary Materials

The following are available online at https://www.mdpi.com/2073-4360/12/3/537/s1, Figure S1: ^13^C spectrum of 1-octyllithium prepared from (1-octyl)_2_Zn in C_6_D_6_, Figure S2: ^1^H and ^13^C NMR spectra of 2-ethylhexyllithium prepared from (2-ethylhexyl)_2_Zn in C_6_D_6_, Figure S3: GPC curve after styrene polymerization performed with no addition of PMDTA (Entry 1 in Table 1), Figure S4: GPC curves before and after styrene polymerization, Figure S5: ^1^H NMR spectrum (C_6_D_6_) of the lithium species generated in the pot of “1-octene + n-BuLi + PMDTA” in methylcyclohexane, Figure S6: ^1^H NMR spectrum (C_6_D_6_) of the species generated by quenching the reaction pot of “1-octene + n-BuLi + PMDTA” in methylcyclohexane with H_2_O or D_2_O, Figure S6: ^1^H NMR spectrum (C_6_D_6_) of the species generated by quenching the reaction pot of “1-octene + n-BuLi + PMDTA” in methylcyclohexane with H_2_O or D_2_O, Figure S8: ^1^H spectrum of “*sec*-BuLi + PMDTA” in C_6_D_12_ (30 min), Figure S9: ^1^H spectrum of C6D5Li × (PMDTA) prepared in the reaction pot of “n-BuLi + PMDTA” in C_6_D_6_, Figure S10: ^1^H spectrum (C_6_D_6_) of the lithium species in the pot of “n-BuLi + PMDTA” in 1-octene, Figure S11: GPC curves before and after styrene polymerization, Figure S12: DSC thermogram of PO-*block*-PS (Entry 5 in Table 3).
