# Supplementary material for: Polystyrene Chain Growth Initiated from Dialkylzinc for Synthesis of Polyolefin-Polystyrene Block Copolymers
Source: Polymers (Basel). 2020 Mar 2;12(3):537. doi: 10.3390/polym12030537 (PMC7182881; doi:10.3390/polym12030537)

Supplementary Materials: Polystyrene Chain Growth from Di-end-functional Polyolefins for Polystyrene-Polyolefin-Polystyrene Block Copolymers

Tae Jin Kim, Jun Won Baek, Seung Hyun Moon, Hyun Ju Lee, Kyung Lee Park, Sung Moon Bae, Jong Chul Lee, Pyung Cheon Lee, and Bun Yeoul Lee*

Department of Molecular Science and Technology, Ajou University, Suwon 443-749, South Korea.

* Correspondence: bunyeoul@ajou.ac.kr; Tel: 82-31-219-1844

Figure S1. ^{13}C spectrum of 1-octyllithium prepared from $(1\text{-octyl})_2Zn$ in C_6D_6

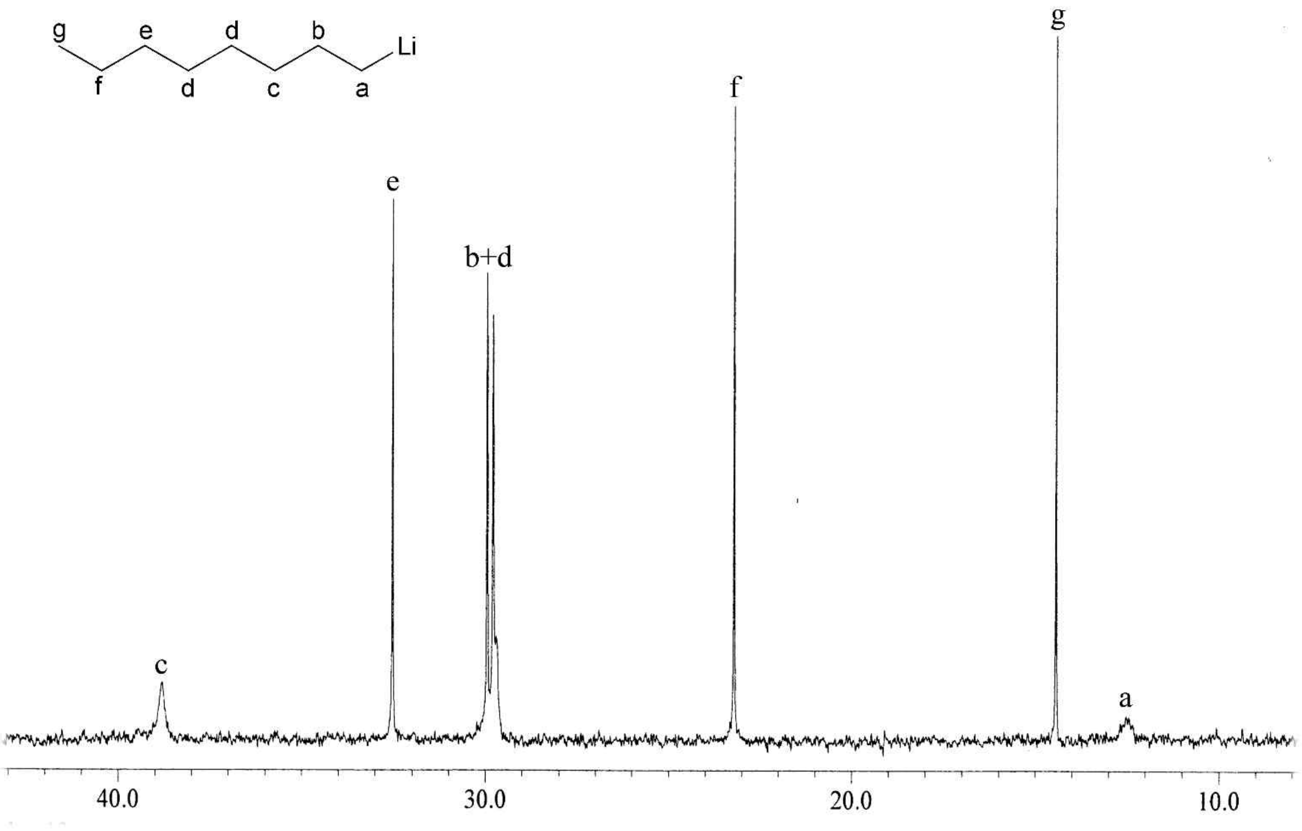

Figure S2. ^{1}H and ^{13}C NMR spectra of 2-ethylhexyllithium prepared from (2-ethylhexyl)$_2$Zn in C_6D_6

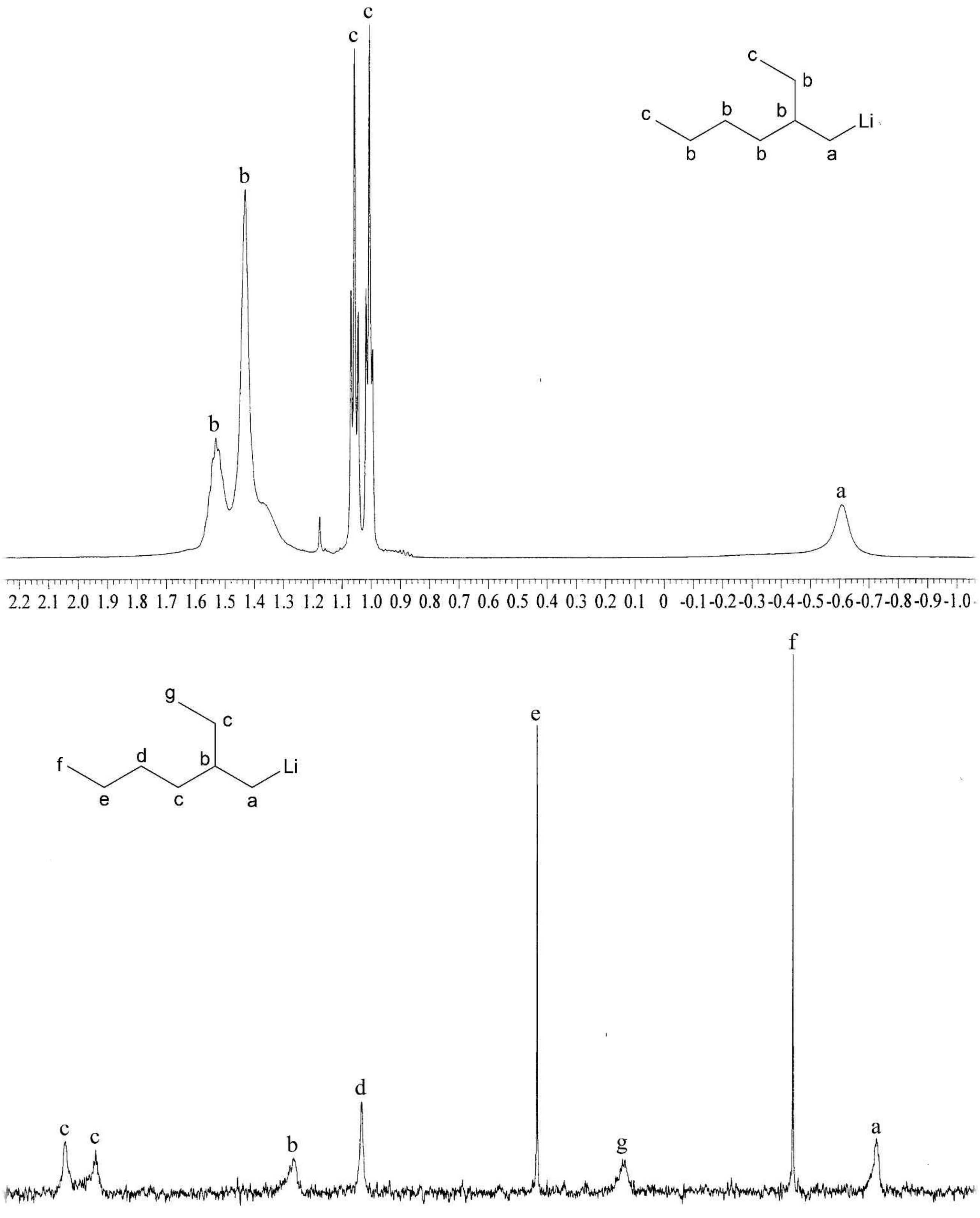

Figure S3. GPC curve after styrene polymerization performed with no addition of PMDTA (Entry 1 in Table 1)

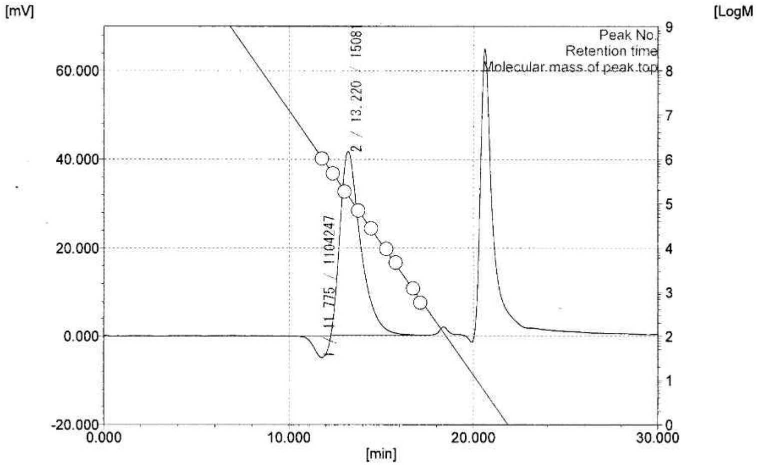

Figure S4. GPC curves before and after styrene polymerization

<Entry 2 in Table 1>

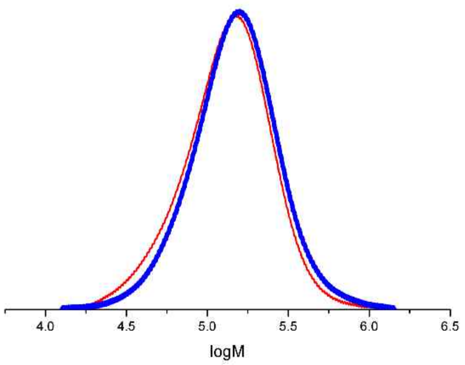

<Entry 3 in Table 1>

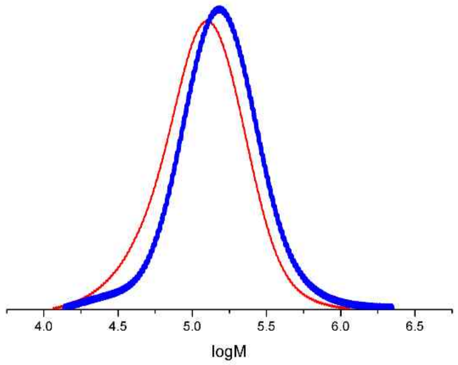

<Entry 4 in Table 1>

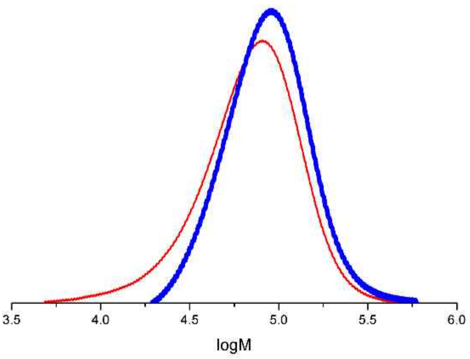

<Entry 5 in Table 1>

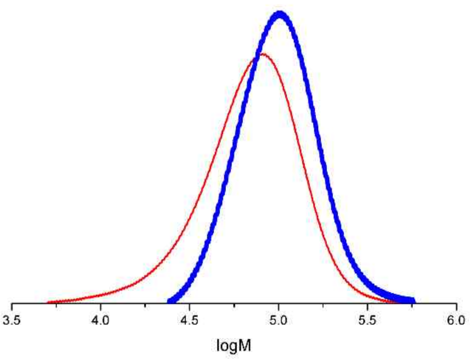

Figure S5. ^{1}H NMR spectrum (C_6D_6) of the lithium species generated in the pot of "1-octene + n-BuLi + PMDTA" in methylcyclohexane

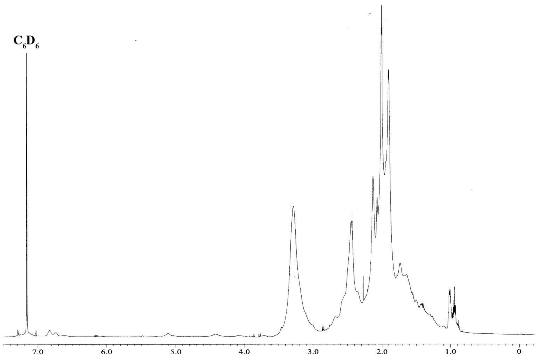

Figure S6. ^{1}H NMR spectrum (C_6D_6) of the species generated by quenching the reaction pot of "1-octene + n-BuLi + PMDTA" in methylcyclohexane with H_2O or D_2O

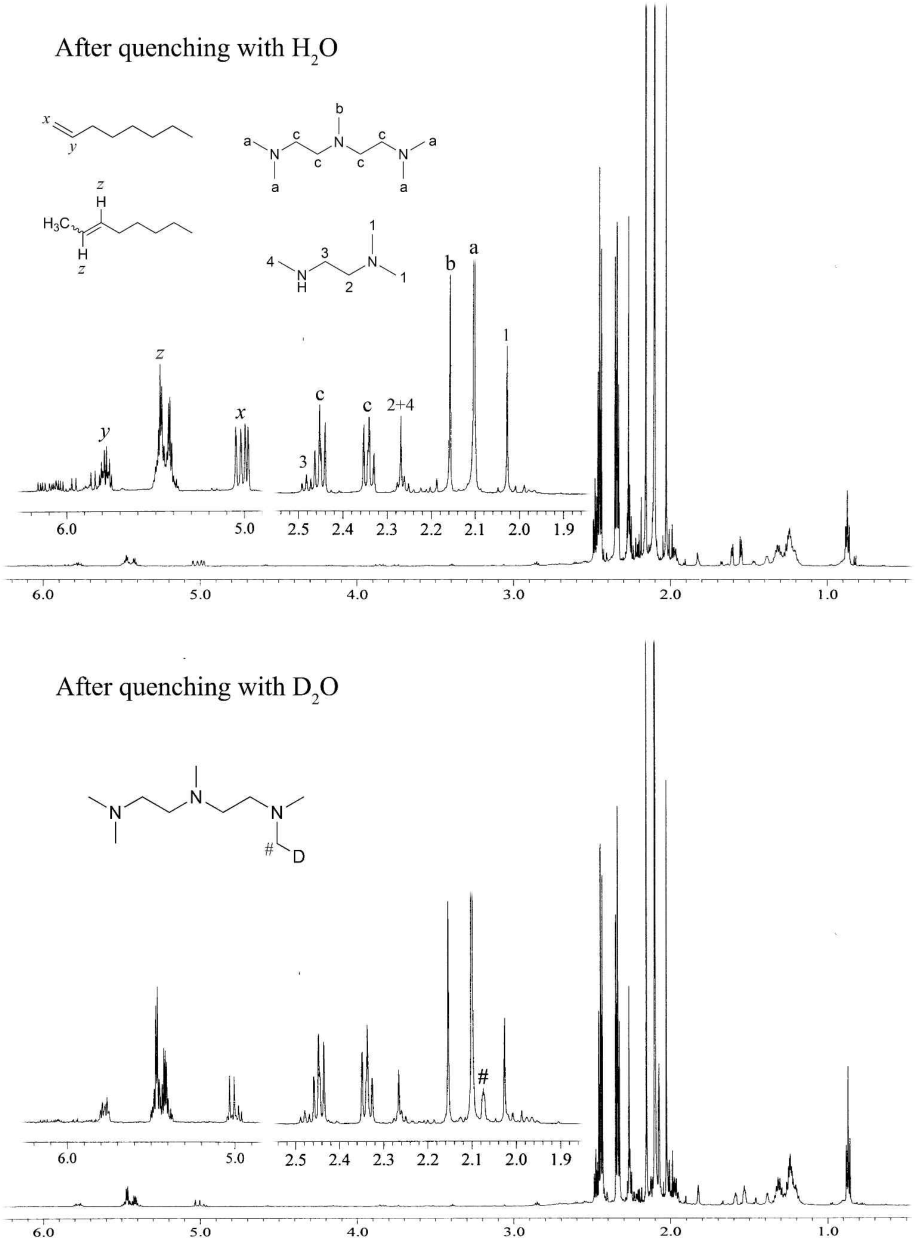

Figure S7. ^{1}H NMR spectra of "n-BuLi + PMDTA" in C_6D_{12}

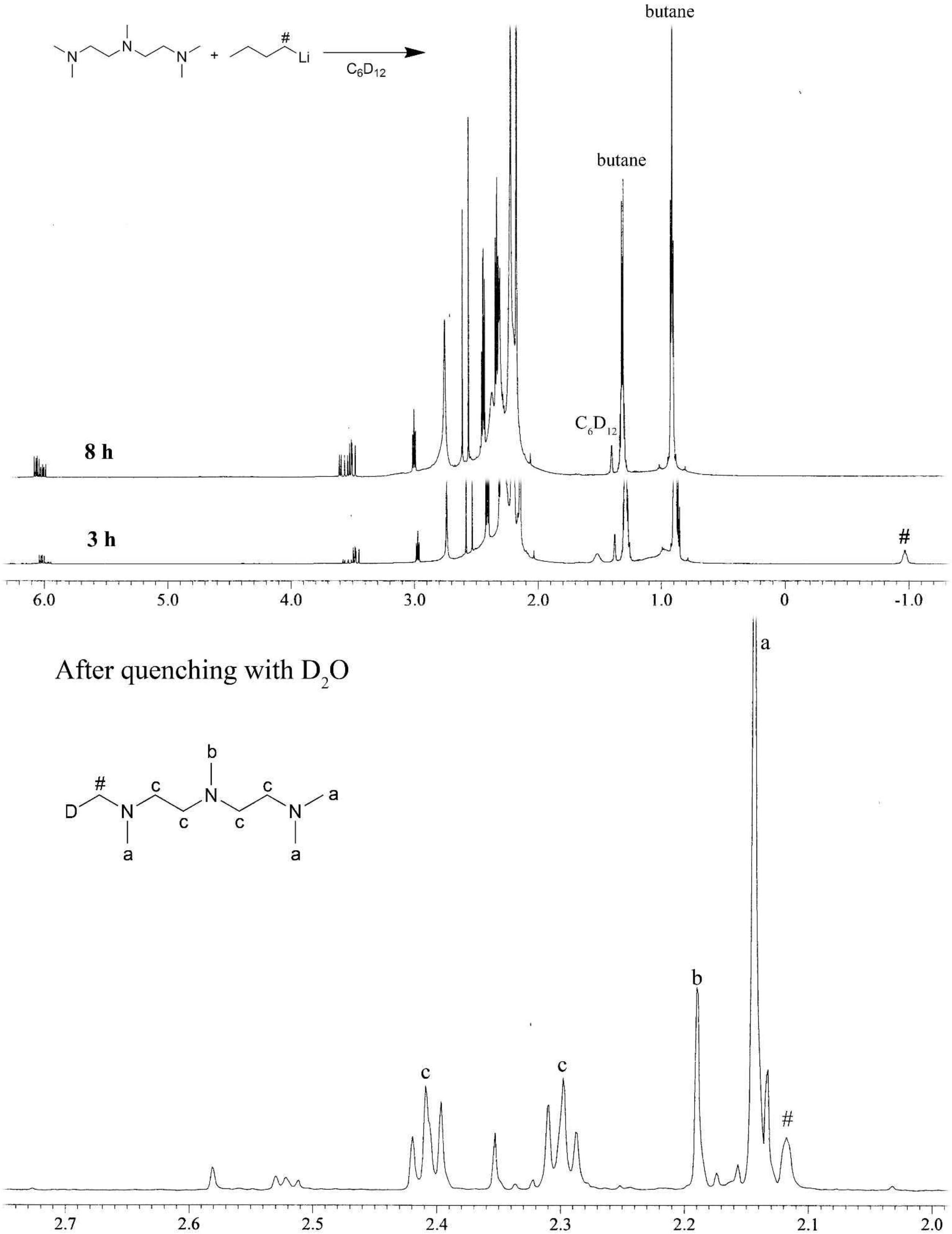

Figure S8. ^{1}H spectrum of "*sec*-BuLi + PMDTA" in C_6D_{12} (30 min).

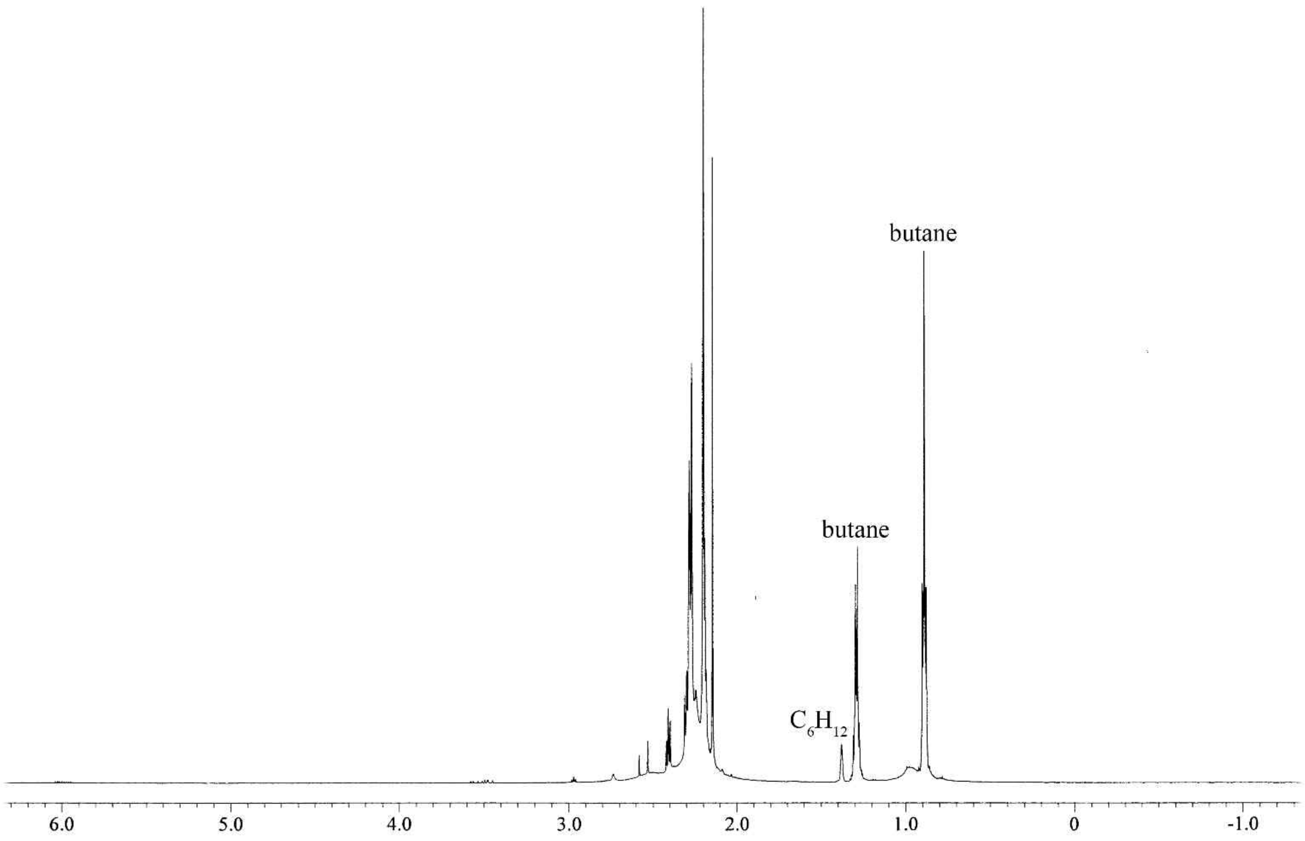

Figure S9. ^{1}H spectrum of $C_6D_5Li{\cdot}(PMDTA)$ prepared in the reaction pot of "n-BuLi + PMDTA" in C_6D_6

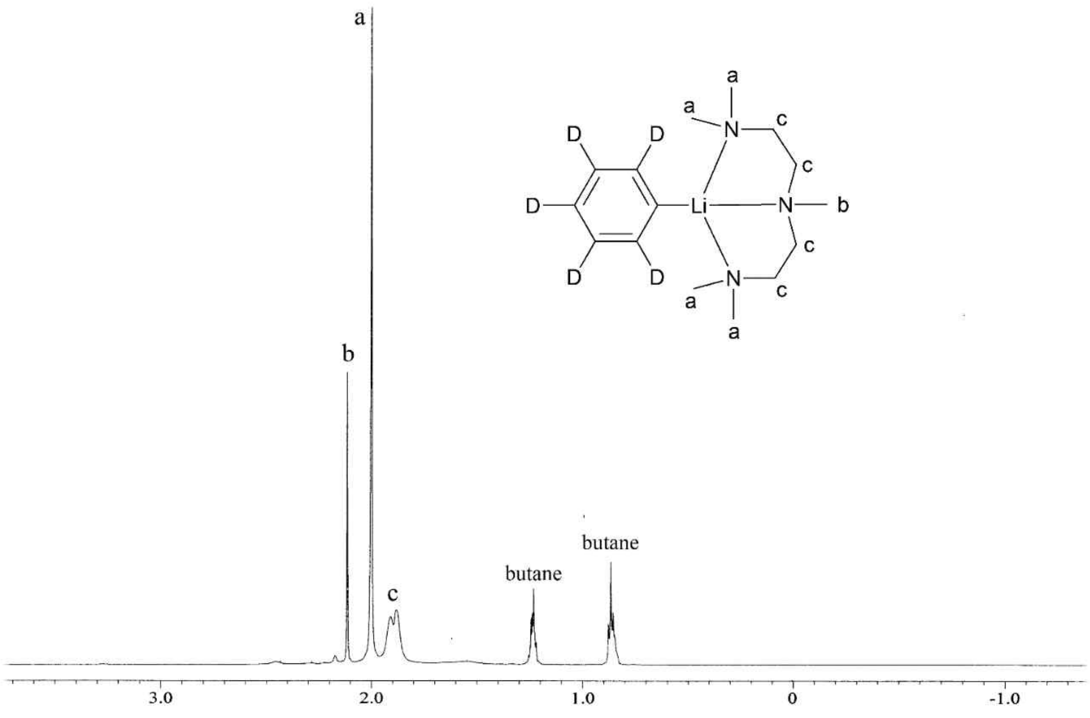

Figure S10. ^{1}H spectrum (C_6D_6) of the lithium species in the pot of "n-BuLi + PMDTA" in 1-octene

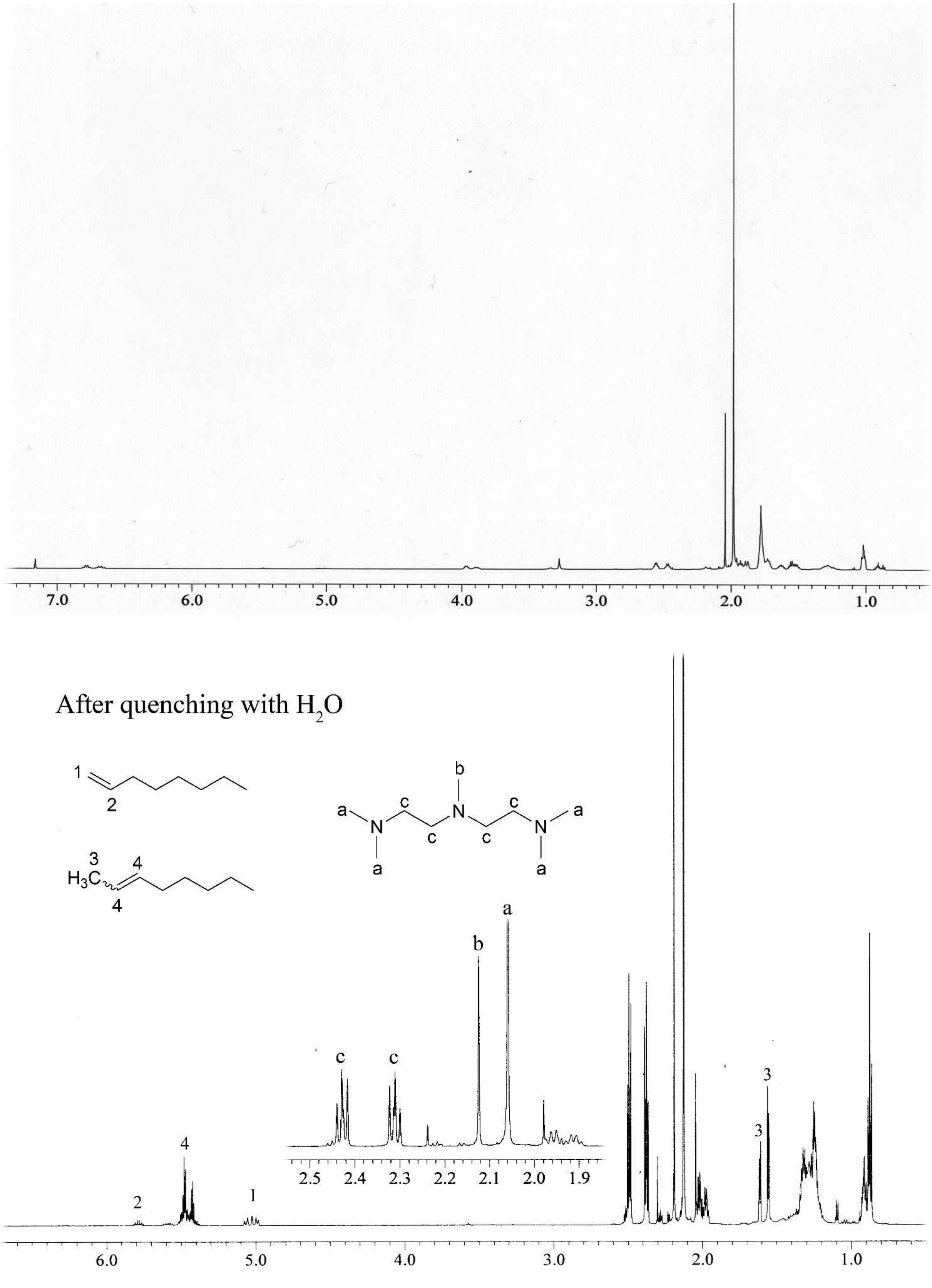

Figure S11. GPC curves before and after styrene polymerization

<Entry 6 in Table 3>

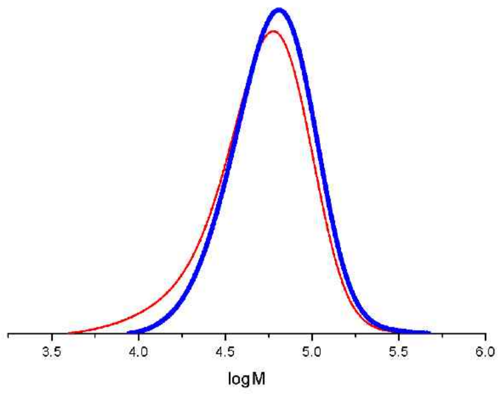

<Entry 7 in Table 3>

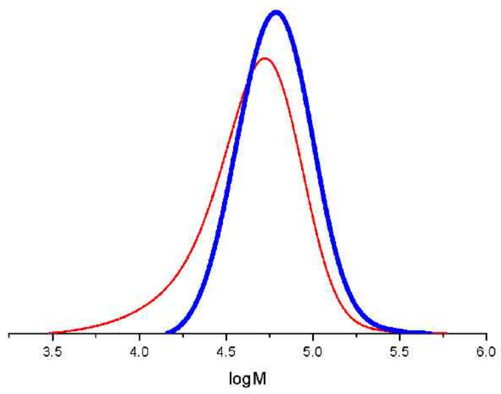

<Entry 8 in Table 3>

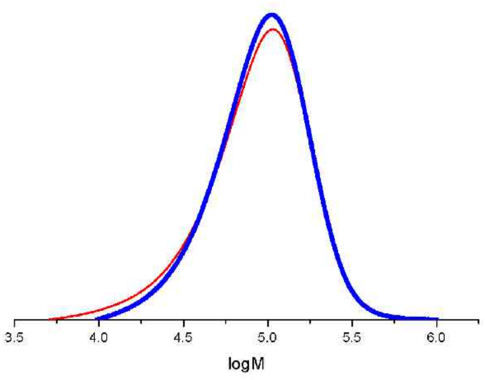

<Entry 9 in Table 3>

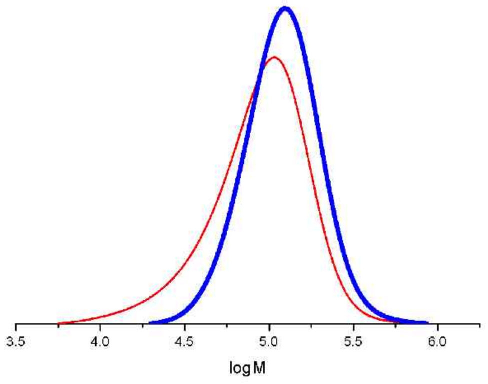

<Entry 10 in Table 3>

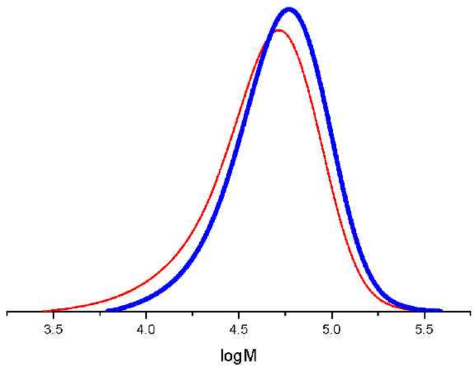

<Entry 11 in Table 3>

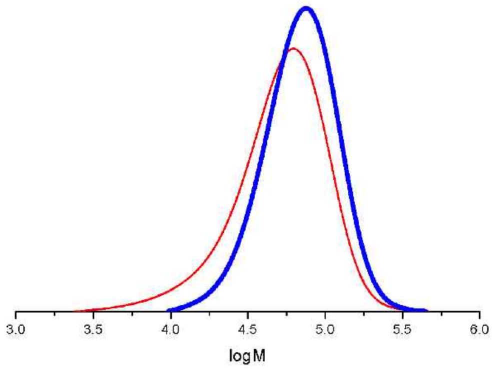

Figure S12. DSC thermogram of PO-*block*-PS (Entry 5 in Table 3)

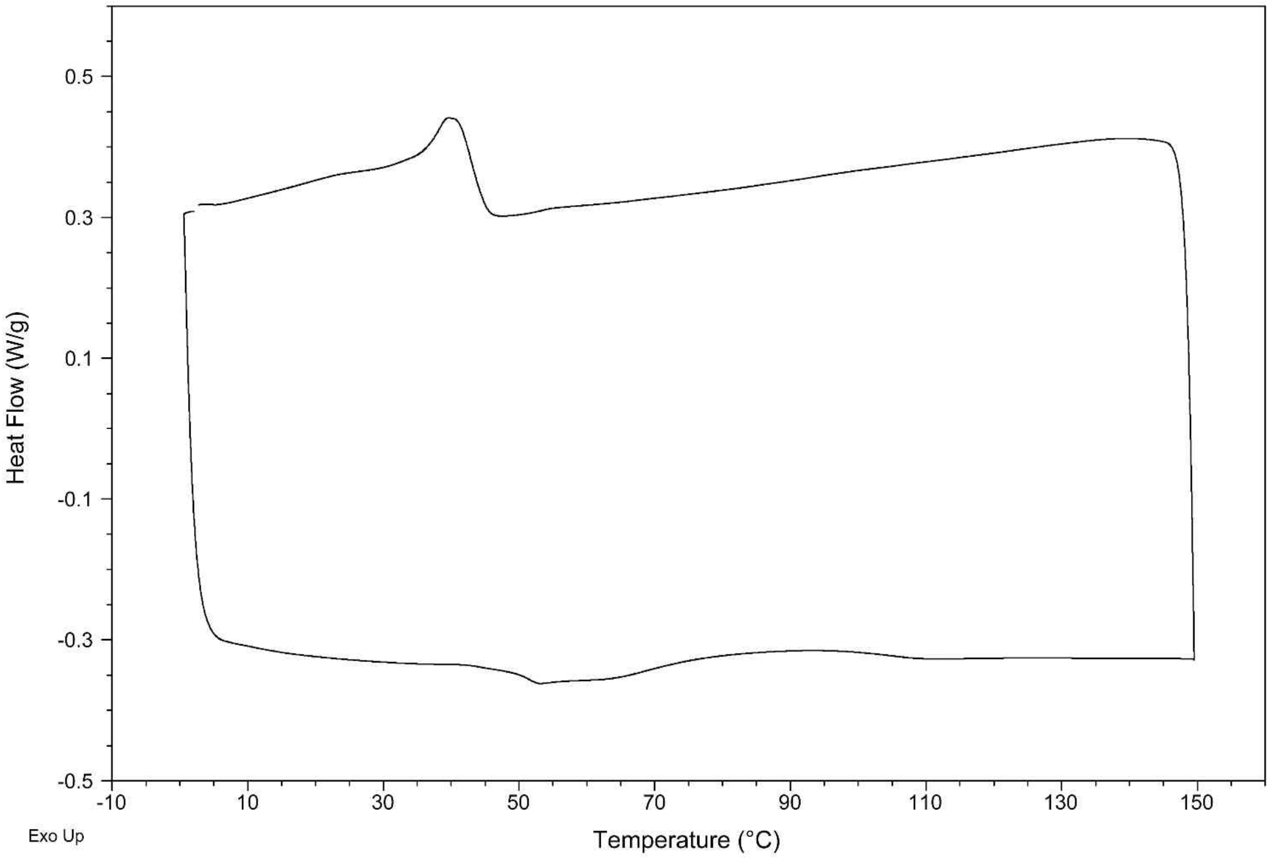

Supplement: Supplementary file 1 [file polymers-12-00537-s001.pdf]
